# The transcription factor ATF5: role in cellular differentiation, stress responses, and cancer

**DOI:** 10.18632/oncotarget.21102

**Published:** 2017-09-20

**Authors:** Thomas K. Sears, James M. Angelastro

**Affiliations:** ^1^ Department of Molecular Biosciences, University of California, Davis School of Veterinary Medicine, Davis, 95616 CA, USA

**Keywords:** CP (cell penetrating peptide), ATF5, CP-d/n-ATF5 (cell penetrating peptide dominant negative ATF5), ER stress, protein homeostasis

## Abstract

Activating transcription factor 5 (ATF5) is a cellular prosurvival transcription factor within the basic leucine zipper (bZip) family that is involved in cellular differentiation and promotes cellular adaptation to stress. Recent studies have characterized the oncogenic role of ATF5 in the development of several different types of cancer, notably glioblastoma. Preclinical assessment of a systemically deliverable dominant-negative ATF5 (dnATF5) biologic has found that targeting ATF5 results in tumor regression and tumor growth inhibition of glioblastoma xenografts in mouse models. In this review, we comprehensively and critically detail the current scientific literature on ATF5 in the context of cellular differentiation, survival, and response to stressors in normal tissues. Furthermore, we will discuss how the prosurvival role of ATF5 aides in cancer development, followed by current advances in targeting ATF5 using dominant-negative biologics, and perspectives on future research.

## Basic molecular and structural properties of ATF5

The transcription factor ATF5 belongs to the bZip family of transcription factors and shares this family with other notable proteins such as cAMP response element-binding protein (CREB), Fos proto-oncogene (FOS), and nuclear factor erythroid-devoid 2-related factor 2 (NRF2). These bZip proteins are composed of an amphipathic leucine zipper that mediates hetero- and homodimerization via a coiled-coil domain along with a basic N-terminal portion involved in DNA binding [1, 2]. ATF5 is classified based on the dimerization properties of its leucine zipper domain into the ATF4 subfamily of bZip transcription factors [3]. As opposed to FOS which primarily heterodimerizes or CREB which primarily homodimerizes, ATF5 is suggested to readily hetero- and homodimerize with transcription factors and is perceived to be quite promiscuous in its binding capabilities [3], though little has been published on ATF5 dimerization partners. With this in mind, one proposed binding partner for ATF5 includes CCAAT/enhancer-binding protein-gamma (C/EBPγ) [4]. Furthermore, ATF5 shows pronounced sequence homology with ATF4 and shares similar modes of regulation and downstream effects i.e. both are regulated translationally via stress-responsive inhibitory uORFs and modulate processes involved in tissue development, apoptosis, and cancer [5–9]. The association between ATF4 and ATF5 function doesn't stop there; it has been found that ATF4 is required for ATF5 gene expression in mouse embryonic fibroblasts [6].

Transcription of the *ATF5* gene results in two mRNA splice variants denoted *ATF5*α and *ATF5*β which harbor unique 5′-UTRs (5′-UTRα and 5′-UTRβ) involved in repression of ATF5 translation. For *ATF5*α, this repression is due to the presence of a conserved upstream open reading frames (uORFs) within the 5′-UTRα, thereby resulting in out of frame transcription regarding the ATF5 coding DNA sequence (CDS) [6, 10]. The two *ATF5* mRNA transcripts are regulated via different mechanisms i.e. amino acid limitation and arsenite exposure release 5′-UTR-mediated repression of *ATF5*α but not *ATF5*β, suggesting the *ATF5*α transcript is preferentially upregulated during stress. It has also been reported that the 5′-UTRα facilitates nonsense-mediated decay of the *ATF5*α transcript [11]. Furthermore, the *ATF5*α transcript was shown to be highly expressed late in gestation and in the liver of adult mice [12]. eIF2α phosphorylation, which generally occurs during cellular stress, drives translation of *ATF5*α mRNA by shifting ribosomal initiation towards the ATF5 CDS and away from the inhibitory uORF.

Control of ATF5 protein expression is governed through proteasomal degradation carried out by Cdc34 and Rad6 E3 ligases [13], with which Cdc34 pathway for ATF5 is inhibited by cisplatin [14]. Other non-stress-related cellular and tissue specific regulatory pathways for ATF5 are discussed in the section below.

## Role of ATF5 in cellular differentiation and tissue development

Numerous studies have reported that ATF5 plays a critical role in modulating differentiation pathways in a variety of tissues such as the brain [15], bone [16], liver [17], and fat [18]. Among these, ATF5-mediated differentiation of neural tissues has been most well characterized. In general, ATF5 appears to hinder differentiation in brain and bone tissues while conversely stimulating differentiation in the liver. This property of ATF5, to modulate tissue-specific differentiation, highlights the challenges in predicting ATF5 activity based on cell type. Because transcription factor activity is greatly influenced by the presence of dimerization partners, this indicates that there may be a wide variety of ATF5-interacting transcription factors that can alter the function of ATF5 based on their tissue-specific expression profiles. Studies utilizing conditional knockouts to evaluate the role of ATF5 in modulating tissue-specific differentiation processes have not been performed and would provide valuable insight into ATF5-dependent differentiation processes for specific tissue types. These functions in tissue development, i.e. regulating the growth and differentiation of stem and progenitor cells, also supports the notion that ATF5 activity may play a role in the self-renewal and differentiation of cancer stem cells.

## Neural development

Relative to other tissue types, the role of ATF5 in the development of neural tissue has been widely studied. Of great importance are the studies utilizing ATF5 knockout mice. These studies showed that ATF5 knockout mice exhibited neonatal lethality due to a competitive suckling deficit [19, 20]. Further investigation revealed that ATF5 knockout mice had significantly smaller olfactory bulbs and that this effect was likely related to reduced proliferation of neural progenitors of the subventricular zone. This highlights the importance of ATF5 in neural development and suggests ATF5 plays a significant role in modulating the growth and differentiation of neural progenitors.

Early studies showed that in PC12 primitive neural crest cells, *ATF5* gene transcripts were downregulated more than 25-fold upon NGF-stimulated differentiation [21]. Further investigation by Angelastro et al. revealed that ATF5 is highly expressed by neural progenitor cells in the ventricular zone of the developing rat brain and that ATF5 maintains neural progenitors in the cell cycle [15, 22]. Angelastro et al. also showed that ATF5 mediates differentiation of neural progenitors in developing rat brain telencephalic cell cultures and that ATF5 represses neurite outgrowth of PC12 cells, which is thought to be via repression of cAMP response element transactivation. In addition to the hindrance of neuronal differentiation by ATF5, it was shown that ATF5 represses the differentiation of neural progenitors into oligodendrocytes and astrocytes *in vitro* and *in vivo* [22, 23]. Lastly, Lee et al. reported that ATF5 promotes sonic hedgehog-mediated proliferation of cerebellar granule neural progenitor cells, thereby indicating that ATF5 plays an important role in neural progenitors outside the cerebral cortex [24]. These findings indicate that ATF5 represses differentiation of neural progenitors and that downregulation of ATF5 is required for differentiation of neural progenitors into astrocytes, neurons, and oligodendrocytes. While this is true, later studies revealed that mature neurons can upregulate ATF5 in response to ER stress and that this has a neuroprotective role against apoptotic cell death [25]. This also suggests that ATF5 possesses functional roles in promoting the survival of neurons. This raises questions about potential off-target toxicities that may be associated with targeting ATF5 for anticancer therapies. With this in mind, studies administering dnATF5 to animals have not resulted in toxicity to normal adult organs and tissues, including the CNS [26, 27].

Further studies intended to define the role of ATF5 in the differentiation of neuroglial cell types such as oligodendrocytes and astrocytes. Indeed, Wang et al. showed that ATF5 mediates the survival and differentiation of olfactory sensory neurons (OSNs) using ATF5 knockout mice [20]. Wang et al. reported that ATF5 knockout mice exhibited a substantial increase in mortality at the neonatal stage and that a faulty olfactory system was responsible for this effect. Specifically, Wang et al. identified that ATF5 was highly expressed in mature OSNs and that this expression was required for differentiation, critical for survival, and activated OSN-specific gene expression of mature OSNs. Various publications further support this role for ATF5 by showing *ATF5* is significantly expressed in the olfactory epithelium and that ATF5 knockout mice possess markedly smaller olfactory bulbs compared to wild type mice [10, 16, 17]. These findings are consistent with the accepted prosurvival and antiapoptotic role of ATF5 that is observed in other cell types. Furthermore, the function of ATF5 in promoting differentiation of OSNs is contrary to the differentiation suppressor role of ATF5 in neural progenitor cells of the cerebral cortex and cerebellum. In addition, this suggests that neonatal lethality in mice may be overcome by giving special attention to competitive suckling deficits that arise from an olfactory defect.

## Hepatic differentiation

Interestingly, ATF5 is most highly expressed in the liver and possesses an opposing role in the differentiation of hepatocytes relative to neural progenitors [12]. This effect was first identified by Du et al. upon analyzing differentially expressed genes in primary and fetal human hepatocytes where ATF5 was seen to be significantly upregulated in mature hepatocytes [28]. Du et al. also showed that ATF5 could perform the role of maturation factor in lineage-reprogrammed human embryonic fibroblasts. Work following this study was able to show that ATF5 also acts as a maturation factor in induced pluripotent stem cells [17]. Lastly, ATF5 has been shown to cooperate with the nuclear receptor constitutive androstane receptor (CAR) to transactivate cytochrome P450 2B6 (CYP2B6), giving evidence that ATF5 mediates transcription of proteins in the liver associated with specialized hepatic function [29]. Overall, these findings indicate that ATF5 is upregulated upon differentiation of hepatocytes as opposed to ATF5-mediated differentiation in neural lineages.

## Osteogenesis

Initial studies by Leong et al. with adipose-derived stem cells (ADSCs), i.e. adipose-derived mesenchymal stem cells, found that upon osteogenic differentiation these ADSCs consistently downregulated ATF5 [16]. Leong et al. also showed that ATF5 knockdown sensitized ADSCs to osteogenic stimulation. While these findings provide limited evidence to supporting the repressive role of ATF5 in osteogenic differentiation, they highlight the functional role of ATF5 in modulating the differentiation of a diverse range of tissue types.

## Adipogenesis

The finding that ATF5 modulates osteogenesis in ADSCs led to studies investigating the role of ATF5 in adipogenesis due to the known overlap between osteogenic and adipogenic signaling pathways e.g. insulin-like growth factor (IGF) signaling. Using a yeast two-hybrid assay supplemented with a pulldown assay, Zhao et al. reported that ATF5 physically interacts with C/EBPβ to bind to and activate the C/EBPα promoter, and that this interaction is mediated by p300 [18]. In addition, Zhao et al. showed that knockdown of ATF5 inhibited adipocyte differentiation through the effect of ATF5 on C/EBPα. Interestingly, Zhao et al. reported that ATF5 expression correlated with obesity in both mice and humans. Overall, these findings give evidence for ATF5-mediated adipogenesis and even suggest a potential role for ATF5 in obesity.

## Role of ATF5 in protein homeostasis (Proteostasis) and the integrated stress response (ISR)

**Figure 1:**
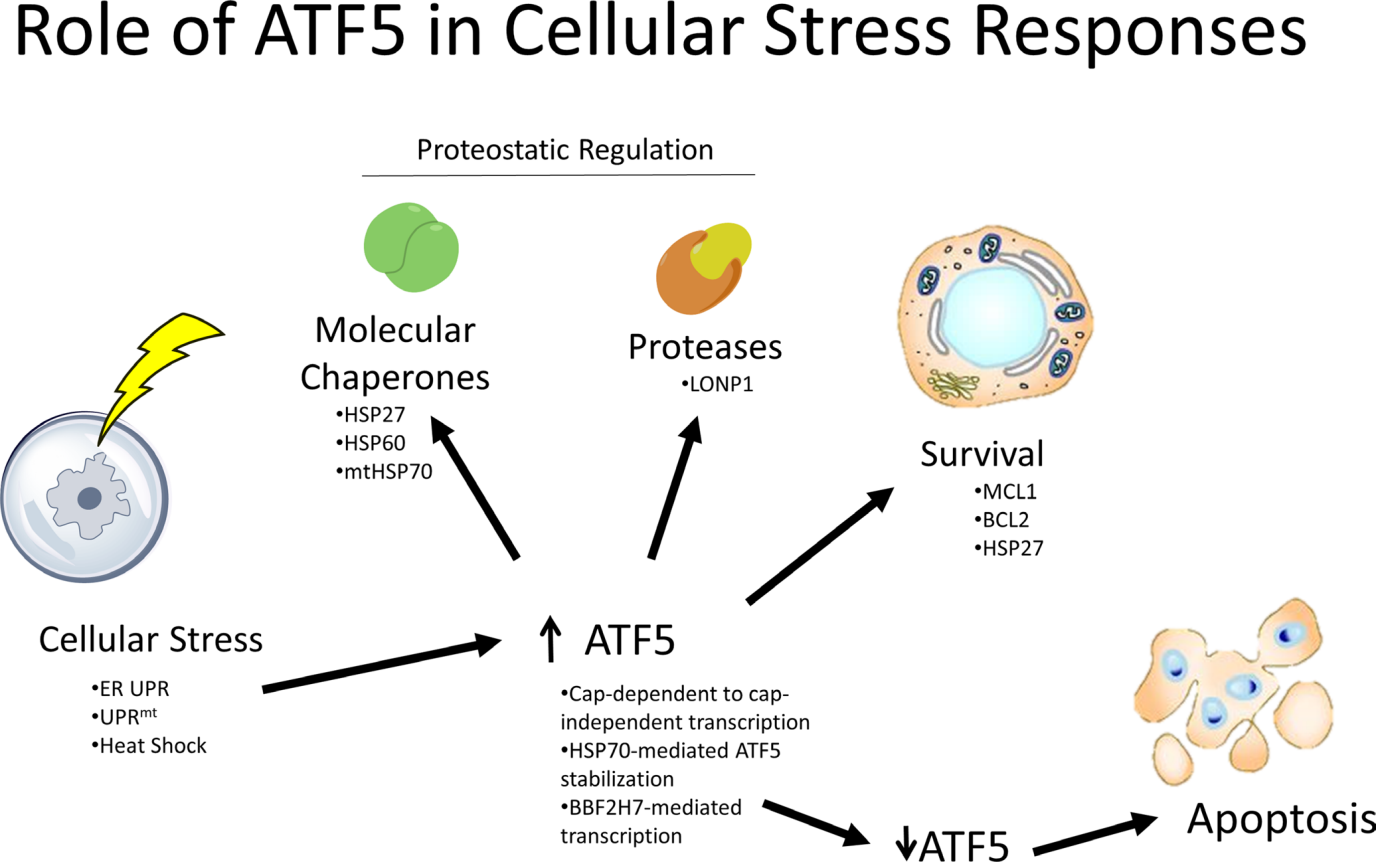
Role of ATF5 in cellular stress responses ATF5 is upregulated by various stressors, which then results in subsequent activation of molecular chaperones, proteases, and prosurvival molecules. Downregulation of ATF5 drives apoptosis in these cells. Image was generated using ChemBioDraw Ultra 14.0 (UC Davis License) and PowerPoint 2013.

ATF5, like other ATF-family transcription factors such as ATF4 and ATF6, mediates transcriptional responses to cellular stressors. Cellular stress responses reported to be influenced by ATF5 activity include the cytosolic heat shock response, the ER unfolded protein response (ER UPR), and the mitochondrial UPR (UPR^mt^). The apparent function of ATF5 in these responses is to mediate transcription of molecular chaperones, proteases, and prosurvival proteins. From this, a simplified model for the role of ATF5 in stress responses can be diagrammed as seen in Figure 1. This Figure exemplifies the function of ATF5 in stress responses where ATF5 is upregulated as transcriptional initiation shifts away from inhibitory uORFs as a component of the ISR [6]. ATF5 can then activate transcription of molecular chaperones and proteases which are involved in restoring proteostasis [30, 31]. If proteostasis is significantly imbalanced, apoptosis can then be triggered by downregulation of ATF5 [25, 32]. It is likely that this prosurvival effect is due to regulation of heat shock proteins and antiapoptotic proteins such as HSP27 [31], BCL-2 [33], and MCL-1 [34], as detailed in the sections below. As most of the mechanistic studies involved with the prosuvival function of ATF5 were done in cancer cells, the mechanism with which ATF5 mediates a prosurvival effect in normal tissues remains to be characterized.

## ER UPR and cytosolic heat shock response

Two specific yet somewhat overlapping cellular responses to stress are the ER UPR and the cytosolic heat shock response. Here, accumulation of misfolded proteins in either the ER or cytosol results in upregulation of chaperones and proteases involved in resolving improper protein folding. Proteasome inhibition and ER stress upregulate ATF5 via PERK-mediated eIF2α phosphorylation, which then results in a decrease in global protein translation and a shift in translational control away from an inhibitory upstream open reading frame (uORF) [6]. This upregulation of ATF5 can then lead to transactivation of HSP27, a molecular chaperone critical in promoting survival and proper protein folding, in response to aberrant protein folding within the cytosol and the ER [31, 35, 36]. In addition, HSP70 has been found to increase ATF5 protein stability, which suggests ATF5 functions to control cell fate in response to perturbations in proteostasis in the context of the cytosolic heat shock response [37]. It was found that in growth plate cartilage the bZip transcription factor BBF2H7 mediates a prosurvival effect in response to ER stress by activating the ATF5-MCL1 pathway [38]. The prosurvival function of ATF5 in response to ER stress was also seen in cultured adult neurons treated with the ER stress-inducing agent tunicamycin [25]. In this study tunicamycin was shown to selectively elicit profound transcriptional activation of genes involved in ER UPR but not mitochondrial UPR, suggesting this response is ER UPR-specific. It was also found that ATF5 regulates and is regulated by the ER stress-responsive and fate-determinative transcription factor CHOP, thereby giving further evidence that ATF5 interacts with signaling pathways involved in ER stress responses and cell survival [39, 40]. Overall, it appears that ATF5 is not only involved in mediating cell survival in the context of the cytosolic heat shock response and ER stress but also initiating transcriptional responses to resolve these perturbations in proteostasis.

## Pancreatic β-cells and ER stress

Within the pancreatic islets lie β-cells which are critical to maintaining insulin levels and energy anabolism. These β-cells are especially susceptible to many stressors, including ER and oxidative stress, and their ability to resolve these perturbations determines their survival [41]. Initially, it was found that ATF5 is a likely downstream target of the transcription factor PDX1, which mediates β-cell susceptibility to ER stress [42]. Further research by Juliana et al. reported that β-cells induced ATF5 expression upon induction of ER stress, and that knockdown of ATF5 made these cells susceptible to ER stress-induced apoptosis via hindered inhibition of global protein translation [32]. While this is true, the physiological relevance of ATF5 activity to β cell function is unclear since in this study ATF5 knockout mice showed no alteration in their glucose homeostasis phenotype in glucose and insulin tolerance tests. However, β-cell apoptosis was increased in ATF5 knockout mice. Additional studies revealed that ATF5 binds to the promoter and upregulates the protein TXNIP, which is involved in coupling ER stress-inflammasome signaling and regulates β-cell survival, therefore indicating that ATF5 may facilitate crosstalk between ER stress and inflammatory pathways [43]. Overall, these findings indicate that ATF5 plays a critical role in regulating β-cell survival in response to ER stress and indicates that ATF5 may contribute to β-cell dysfunction in diseases such as diabetes mellitus.

## Mitochondrial UPR

With much of the past research focused on evaluating the role of ATF5 in ER stress, a more recent study revealed the importance of ATF5 in mitochondrial stress. Mitochondria possess their own unfolded protein response (UPR^mt^) that is initiated upon accumulation of unfolded protein within the mitochondrial matrix, thereby activating CHOP and C/EBPβ to regulate expression of mitochondrial stress genes [44]. Analysis of autosomal dominant ataxia patients, characterized by mutation in a mitochondrial protease gene, showed a significant induction of ATF5, thus suggesting ATF5 is induced by mitochondrial stress [45]. A recent study by Fiorese et al. showed that ATF5 has many parallel functions relative to the transcription factor ATFS-1 that mediates UPR^mt^s in *C. elegans*. In this study, it was shown that ATF5 can recapitulate the UPR^mt^ in ATFS-1 KO *C. elegans* by upregulating genes involved in resolving UPR^mt^s such as mitochondrial chaperones and proteases [30]. Fiorese et al. reported the presence of a mitochondrial targeting sequence (MTS) within ATF5 and that in *C. elegans* ATF5 localizes to mitochondria in the absence of stress and moves to the cytosol/nucleus during mitochondrial stress, thereby indicating that ATF5 mediates UPR^mt^ via organelle partitioning. Lastly, Fiorese et al. reported that in mammalian cells ATF5 mediates maintenance and recovery of mitochondrial function after induction of UPR^mt^ by regulating cell viability and transcription of mitochondrial chaperones and proteases. These chaperones and proteases include HSP60, mtHSP70, and LONP1. While this research provides a novel function for ATF5 in cellular stress responses, it imposes further curiosity whether the UPR^mt^ processes mediated by ATF5 can contribute to cancer development in ATF5-dysregulated cancers [46]. With this in mind, ATF4 has also been shown to regulate mitochondrial stress responses [7] and ATF4 and ATF5 are paralogs of ATFS-1. Therefore, the function of ATFS-1 in C. elegans may represent the activity of both ATF4 and ATF5 [47].

## Role of ATF5 in the development of a diverse range of cancers

**Figure 2:**
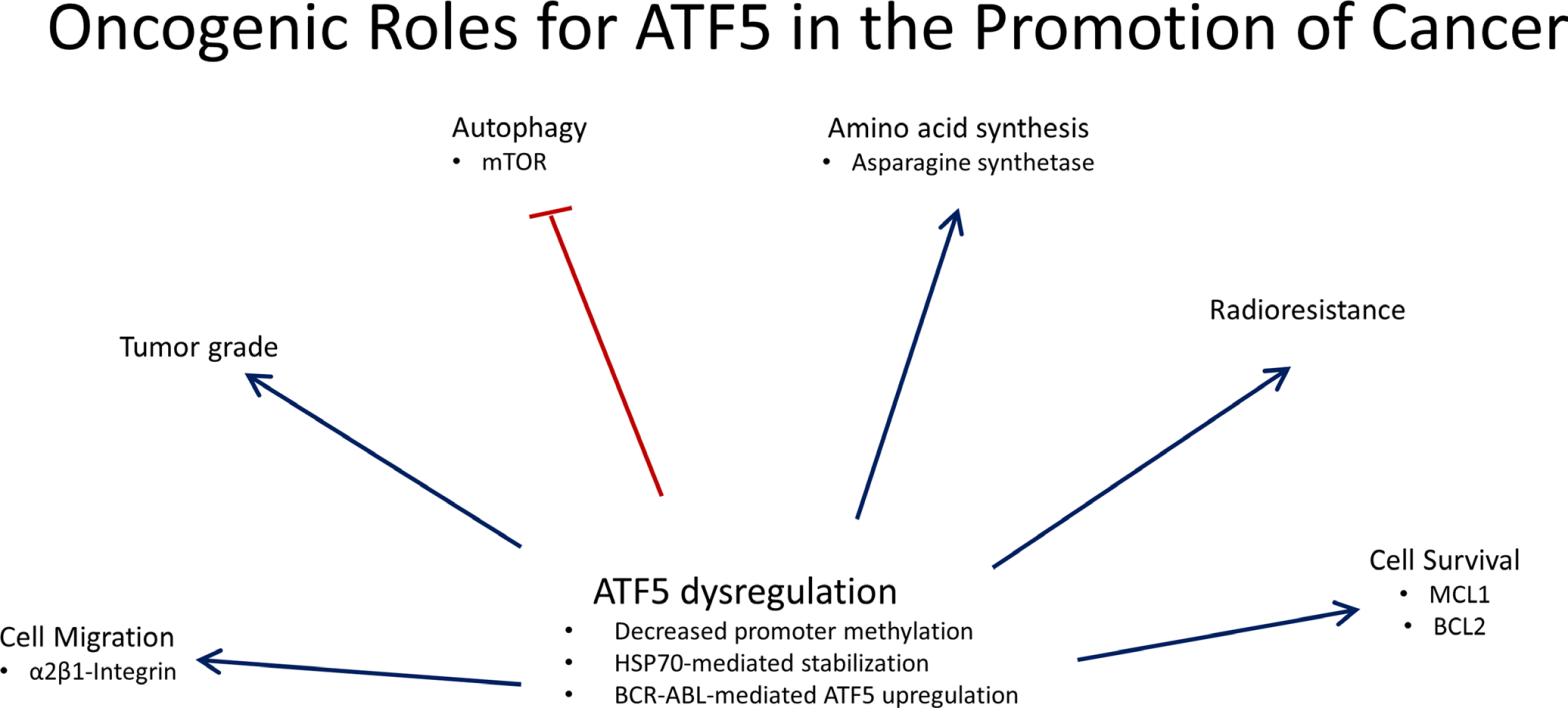
Oncogenic roles for ATF5 in the promotion of cancer ATF5 is dysregulated in a variety of cancer types and is associated with effects on cell migration, amino acids synthesis, radioresistance, and cell survival while inhibiting autophagy. ATF5 is also associated with tumor pathology where ATF5 expression is positively correlated with tumor grade. Image was generated using PowerPoint 2013.

With the role of ATF5 in promoting survival in normal cells established [31, 33, 37], it could be hypothesized that dysregulation of ATF5 may serve as a survival factor in cancer. Indeed, upon analyzing the expression levels of ATF5 in a variety of cancer types it is evident that ATF5 expression is highly upregulated in various forms of cancer such as glioma, breast cancer, lung cancer, and numerous others. This suggests an oncogenic role for ATF5 in these cancer types where overexpression is seen in malignant tissues. The oncogenic functions for ATF5 are outlined in Figure 2, where ATF5 has been shown to promote cell survival, migration, radioresistance, and amino acid synthesis while inhibiting autophagy. It could be hypothesized that these functions mediated by ATF5 in cancer also occur in nontransformed cells, but this hypothesis has yet to be tested. ATF5 expression is also significantly associated with tumor grade in several cancer types [48, 49]. In addition to these functions is the novel role for ATF5 as a structural protein where it facilitates formation of the centrosome, thereby suggesting ATF5 may promote proper mitotic function [50]. Although this is intriguing, no studies have been conducted assessing whether ATF5 promotes cancer development via its centrosomal function. Another interesting property for ATF5 in cancer is seen in hepatocellular carcinoma where ATF5 appears to possess a tumor suppressor role [51].

## Glioma

Of the cancers with which the role of ATF5 has been investigated, gliomas have been the most extensively studied. Initial expression profiling of glioblastoma (GBM) revealed ATF5 expression was inversely correlated with overall patient survival, indicating the importance of ATF5 expression to patient outcomes in the clinic [34, 52]. ATF5 expression was also shown to be upregulated in GBMs and anaplastic gliomas relative to low grade gliomas and normal cortical tissue, indicating that ATF5 expression may be associated with more malignant glioma phenotypes [53]. Indeed, analysis of ATF5 promoter methylation in glioma revealed that there is a substantial decrease in promoter methylation in high grade gliomas relative to low grade gliomas and normal tissue, thereby suggesting alterations in promoter methylation drive aberrant ATF5 expression in high grade gliomas [49]. In addition, ATF5 was shown to mediate the survival response to serum deprivation and staurosporine treatment in C6 glioma cells supporting the notion that ATF5 has an oncogenic and prosurvival role in gliomas [33].

Interference with ATF5 function by transient transfection of a dominant-negative elicited an apoptotic response in glioma cell lines but spared astrocytes, though astrocytes ectopically expressing dnATF5 exited the cell cycle [54]. Further studies were performed using a tetracycline-inducible Tet-off mouse model to induce expression of dnATF5 driven by a GFAP promoter, and retrovirally-induced brain tumors were shown to be regressed and prevented by doxycycline withdrawal [27]. These results led to the development of a systemically deliverable dnATF5 peptide fused to a penetratin motif to allow for extracellular entry [55, 56]. A recombinant FLAG-tagged version of this peptide was shown to effectively cross the blood-brain barrier after IP injection, elicit apoptosis within the tumors, and fully eradicate virally-induced brain tumors [55]. A chemically synthesized variant of this dominant-negative peptide, termed CP-d/n-ATF5-S1, was shown to elicit a significant apoptotic response in culture and inhibit growth of treatment-resistant glioblastoma xenografts, though tumor regression was not observed [56]. These findings point to the significance of ATF5 in the development of gliomas and suggest that ATF5-targeted therapy is a viable treatment option for glioblastoma, which is one of the deadliest forms of cancer and is characterized by a dismal prognosis [57].

**Figure 3:**
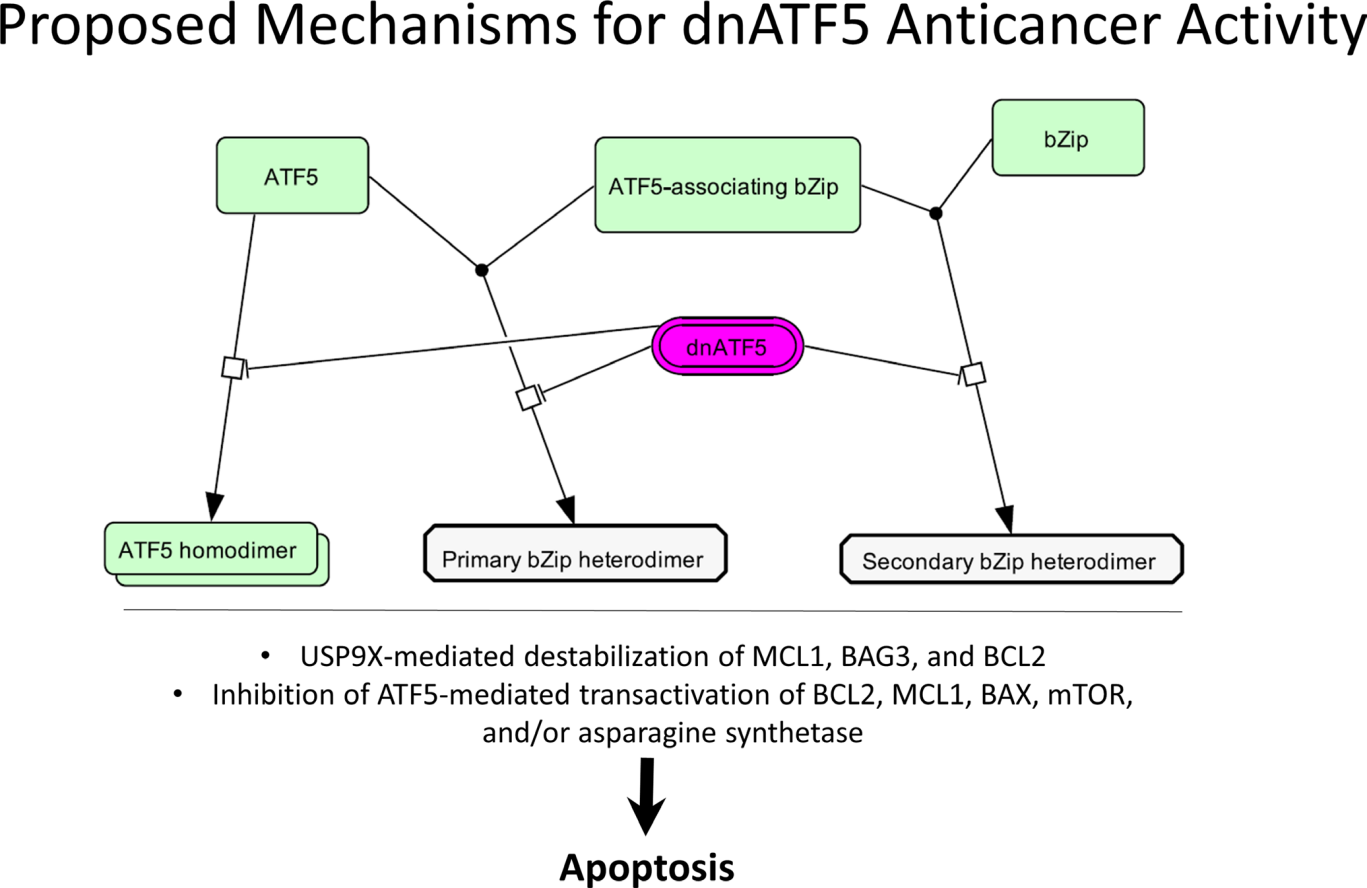
Proposed mechanisms for dnATF5 anticancer activity dnATF5 is thought to disrupt ATF5 homo/heterodimerization or homo/heterodimerization of two non-ATF5-interacting bZip transcription factors. This results in USP9x-mediated destabilization of antiapoptotic proteins or inhibition of ATF5-mediated transactivation of BCL2, MCL1, BAX, mTOR, and asparagine synthetase, thereby leading to apoptosis. Image was generated using CellDesigner 4.4 and PowerPoint 2013.

The mechanisms which ATF5 promotes cell survival have been well studied in gliomas relative to other cancer types. For example, Karpel-Massler et al. showed that interference with ATF5 activity by CP-d/n-ATF5-S1 administration downregulated the antiapoptotic proteins BCL2 and MCL1, though it is suggested that downregulation of these proteins is mediated by effects on the deubiquitinase Usp9x [56]. The proposed paradigm is that Usp9x stabilizes the antiapoptotic proteins BAG3, MCL1, and BCL2, and that CP-d/n-ATF5-S1 mediates downregulation of Usp9x, thereby leading to an apoptotic response. This is further supported by findings that Usp9x knockdown recapitulated the apoptotic effects of CP-d/n-ATF5-S1 and also led to downregulation of BAG3, MCL1, and BCL2 in various glioblastoma cell lines [56]. Though this is true, no experiments have been conducted to evaluate whether the effect of CP-d/n-ATF5-S1 on USP9X is ATF5-specific, and as can be seen in Figure 3, the anticancer mechanism of dnATF5 can be attributed to disruption of ATF5 homodimers, heterodimers, or ATF5-associating bZip homo/heterodimers. Li et al. reported that in C6 glioma cells ATF5 activated transcription of BCL2 in an ATF5-specific response element (ARE)-dependent fashion, though no ChIP assays were performed to assess direct promoter binding [33]. Contrary to these results, Sheng et al. reported that BCL2 expression is not regulated by ATF5 but that ATF5 binds directly to the promoter of MCL1 to activate its transcription [34]. While these results confound one another, the activity of ATF5 in a cell is highly dependent on coexpression with associating transcription factors. Therefore, the effect of ATF5 knockdown can be highly cell type-dependent and could therefore explain why BCL2 seems to be regulated by ATF5 in some glioblastoma cell lines but not in others.

Lastly, Li et al. reported that the molecular chaperone HSP70 was found to stabilize ATF5 protein by physical interaction with the N-terminal proline rich portion of ATF5 [37]. HSP70-mediated stabilization of ATF5 led to increased ATF5 activity and transcription of downstream ATF5 targets such as BCL2. This reveals an important post-translational mechanism with which cancer cells can aberrantly upregulate ATF5 activity by increasing the half-life of ATF5.

## Breast carcinoma

Most of the work on ATF5 in relation to breast cancer has focused on ATF5-mediated promotion of cell survival, though studies have been conducted analyzing ATF5-mediated invasive characteristics. Monaco et al. reported a significant upregulation of nuclear ATF5 expression in breast cancers relative to paired normal breast tissue with a significant upregulation taking place in invasive ductal, invasive lobular, *in situ* ductal, and *in situ* lobular carcinomas [58]. Analysis revealed there was no correlation between ATF5 expression and marker status i.e. ER/PR and HER2/neu. It was also shown by Monaco et al. that upon interference with ATF5 function via use of dnATF5 transgene, breast cancer cell lines underwent apoptosis while nonneoplastic cell lines were spared of this effect. Dluzen et al. showed that in a breast cancer cell line, in response to apoptotic triggering mechanisms of serum deprivation and staurosporine treatment, ATF5 was downregulated. Conversely, forced ATF5 expression blocked apoptosis resulting from serum deprivation and staurosporine treatment. These data suggest that ATF5 acts as an anti-apoptotic transcription factor in breast cancer [33]. This study also reported that ATF5 is responsible for the transactivation of BCL-2 via an ATF5-specific regulatory element, and that this is predominantly responsible for the cell-type dependent antiapoptotic function of ATF5 in breast cancer. Also shown in parallel with glioma, ATF5 activated expression of the early growth response factor 1 (EGR-1) gene in a breast cancer cell line via a novel ATF5 DNA regulatory element [59]. These results give preliminary *in vitro* data supporting ATF5 as a potential target for treatment of breast cancers, but detailed *in vivo* studies probing the function of ATF5 in breast cancer have yet to be performed.

A recent study by Nukuda et al. reported that ATF5 correlated with invasive capability of breast cancer cell lines, and that siRNA knockdown of ATF5 reduced the invasiveness of these cells [60]. In addition, it was shown that ATF5 knockdown resulted in reduced spindle-like morphology and expression of integrins α2β1. These findings indicate that while ATF5 may function to promote cell survival in breast cancer, it may also function to promote metastasis by regulating the invasive characteristics of breast carcinomas. Further studies composed of *in vivo* and clinical studies would help better evaluate the role of ATF5 in regulating the invasive characteristics of breast cancers.

## Leukemia and lymphoma

The importance of ATF5 in the development of leukemia first became evident during analysis of its role in the survival of myeloid progenitor cells. It was found that upon IL-3 deprivation, myeloid progenitor cells underwent apoptosis and subsequent gene expression profiling revealed ATF5 as the most highly downregulated gene [61]. Ectopic expression of ATF5 inhibited the apoptotic response induced by IL-3 deprivation [62]. Sheng et al. reported that BCR-ABL induces ATF5 expression through the PI3K/AKT/FOXO4 pathway, and that ATF5 binds directly to the promoter of mTOR to activate its transcription [63]. In addition, this activation of mTOR suppressed autophagy and it was shown that imatinib treatment induced autophagy at least in part by downregulating ATF5 and the downstream mTOR. These findings suggest that ATF5 expression is a critical mediator of autophagic death in BCR-ABL chronic myelogenous leukemias in response to imatinib treatment.

Several studies have reported the significance of ATF5 activity to clinical outcomes in leukemia patients [64, 65]. Mittal et al. analyzed samples from chronic lymphocytic leukemia (CLL) patients harboring 11q and trisomy 12 chromosomal aberrations and found that these patients had significantly higher expression of ATF5 relative to CLL patients with normal karyotype or 13q deletion [65]. It was also found that these patients with higher ATF5 expression had significantly worse clinical outcomes in the context of time to first treatment.

Rousseau et al. reported that in childhood acute lymphoblastic leukemia (ALL), polymorphisms in the 5′UTR of the *ATF5* transcript altered the expression of *ATF5* and were also associated with event free survival of childhood leukemia patients [64]. Since ATF5 has been shown to regulate asparagine synthetase expression, and asparaginase is a common treatment in childhood ALL, it is possible that interfering with ATF5 pathways in combination with asparaginase treatment could yield better patient outcomes.

Follicular lymphoma (FL), characterized by slow disease progression, often undergoes transformation to aggressive diffuse large B-cell lymphomas (DLBCL) [66]. Bisikirska et al. analyzed gene expression data using the Master Regulator Inference Algorithm (MARINa) and identified ATF5 as one of several transcription factors responsible for transformation of FLs to DLBCLs [66]. This study also found that ATF5 was significantly upregulated in DLBCL compared to FL and that knockdown of ATF5 partially restored the FL expression signature in DLBCL cell lines. These findings suggest that ATF5 expression may be a valuable prognostic marker in lymphomas and that targeting ATF5 or ATF5-mediated pathways may provide therapeutic benefit to patients with DLBCLs.

## Lung carcinoma

Previous research by Ishihara et al. has helped clarify the role of ATF5 in the promotion of lung cancer via modulating the growth and invasiveness of A549 lung carcinoma cells [67]. Ishihara et al. showed that A549 cells with stable ATF5 transfection were resistant to gamma irradiation and that ATF5 expression was highest during the G1/S transition through the cell cycle. A549 cells were exposed to gamma irradiation at different times post-cell cycle synchronization and cells at the G1/S boundary exhibited higher radioresistance, thereby indicating that radioresistance correlates with cell cycle-dependent expression of ATF5. Cyclin levels of ATF5 transfected cells were also significantly altered, with cyclin A2 being upregulated and cyclin E1 downregulated. This, together with an enhanced growth rate *in vitro* and *in vivo* of ATF5-transfected cells, suggests ATF5 promotes cell cycle progression and growth of lung carcinomas.

Ishihara et al. also looked at the effect ATF5 has on the invasive phenotype of A549 cells. ATF5-transfected A549 cells showed an enhanced migratory phenotype with ATF5 siRNA knockdown abrogating this promigratory effect. This promigratory effect of ATF5 was also shown to be reliant on ATF5-dependent induction of β1 integrin. In addition to ATF5 modulating growth and invasiveness of a lung cancer cell line, Ishihara et al. analyzed clinical lung cancer data from various cancer genome databases and showed that ATF5 expression correlated well with overall patient survival. Therefore, ATF5 is suggested to facilitate lung cancer development by promoting cellular proliferation and radioresistance while also being associated with poorer patient prognosis. It would be interesting to know if the expression levels of ATF5 in the ATF5-transfected A549 cells in this study are comparable to expression levels in clinical lung carcinoma samples.

Lastly, Fernandez et al. show via global gene expression analysis that loss of the tumor suppressor LKB1/STK11 leads to significant upregulation of ATF5 in primary lung carcinomas [68]. Though this is intriguing, the role that ATF5 plays in promoting LKB1/STK11 mutant lung carcinomas is unknown.

## Ovarian carcinoma

Chen et al. analyzed clinical samples of epithelial ovarian carcinomas for ATF5 expression and saw a significant upregulation of ATF5 compared to benign and normal ovarian tissue, with ATF5 expression also correlating to cancer stage [69]. In addition, interference with ATF5 activity by transient transfection of dnATF5 elicited a substantial apoptotic response that was accompanied by a downregulation in BCL2 expression. There was no investigation into whether BCL-2 downregulation was responsible for the apoptotic effect and whether there were any ATF5-independent effects of the dnATF5.

## Pancreatic carcinoma

ATF5 expression has been confirmed in pancreatic cancer cell lines and significant upregulation is seen in primary tumors relative to normal pancreatic tissue, suggesting a role for ATF5 in the development of pancreatic cancer [70]. Hu et al. investigated the role that ATF5 plays in mediating pancreatic cancer resistance to paclitaxel chemotherapy. It was shown that interfering with ATF5 function by use of a dnATF5 protein elicited a significant apoptotic response in SW1990 pancreatic cells, and that combination treatment with paclitaxel further enhanced paclitaxel-induced apoptosis. In addition, ATF5 was shown to induce and inhibit promoter activity of BCL-2 and BAX, respectively, with ectopic expression of ATF5 also abrogating paclitaxel-induced downregulation of the BCL-2 promoter. These findings suggest that the activity of ATF5 mediates survival of pancreatic cancers via regulation of BCL-2 and BAX, and that interference with ATF5 activity potentiates apoptosis mediated by paclitaxel treatment. While this is significant, the studies were performed with a single cell line and therefore more studies need to be conducted to establish the association between ATF5 activity and clinical presentations of pancreatic cancer.

Karpel-Massler et al. evaluated the effect of a synthetic cell-penetrant variant of dnATF5 (CP-d/n-ATF5-S1) on the growth of a diverse panel of treatment resistant cancers, including the PANC-1 pancreatic carcinoma cell line [56]. These cells elicited a robust *in vitro* response to CP-d/n-ATF5-S1 administration, showing a significant dose-dependent decrease in cell viability and increase in apoptosis. Notably, at the highest dose of CP-d/n-ATF5-S1 the early apoptotic response in PANC-1 cells reached over 60% of the cell population while late apoptosis/necrosis consisted of about 20%. The antiapoptotic proteins MCL-1 and BCL-2 were also shown to be significantly downregulated upon administration of CP-d/n-ATF5-S1 in a concentration-dependent fashion. The proapoptotic effect of BH3 mimetics, with which MCL-1 mediates apoptotic resistance, was shown to be significantly augmented by combination treatment with CP-d/n-ATF5-S1. Interestingly, the growth of PANC-1 xenografts in the flank of mice were not significantly altered in response to intraperitoneal CP-d/n-ATF5-S1 treatment while many other tumor types showed significant growth reduction. There was no subsequent investigation into the reason for this lack of response, and reduced *in vivo* tumor uptake of CP-d/n-ATF5-S1 was not evaluated.

## Rectal carcinoma

Though little is known about the role of ATF5 in the development of rectal carcinomas, Kong et al. showed that ATF5 protein levels are significantly upregulated in rectal carcinomas relative to normal rectal tissues [48]. Interestingly, in this study ATF5 mRNA levels between neoplastic and nonneoplastic tissues were not significantly different, therefore indicating that upregulation of ATF5 protein levels are likely mediated via post-transcriptional mechanisms in rectal cancers [48]. Additionally, positive ATF5 staining was significantly associated with poorly differentiated rectal tumors relative to moderate-well differentiated tumors [48]. These findings indicate ATF5 plays a role in the development of rectal cancers and that high expression of ATF5 is associated with greater tumor grade.

## Renal cell carcinoma

ATF5 expression has been confirmed in a variety of renal cell carcinoma (RCC) cell lines and ATF5 expression was shown to be significantly upregulated in RCC tissues, though these results were limited by the fact the researchers only tested a single nonneoplastic kidney cell line [71]. In patient-derived RCC tissue sections, only a third of tissues tested stained positive for ATF5 and there was no significant difference in ATF5 expression compared to nonneoplastic renal tissue [58]. This would suggest ATF5 may not play a significant role in aiding the development of renal cell carcinomas. Conversely, Morris et al. identified ATF5 as a candidate tumor suppressor gene through epigenetic analysis of differential gene expression in RCC cells treated with a demethylating agent [72]. ATF5 was significantly upregulated in several RCC cell lines after DNA demethylation, indicating a potential tumor suppressor role for ATF5 in RCC, perhaps similar to the role of ATF5 in hepatocellular carcinoma. Upon a more extensive analysis of RCC cell lines, it was shown that ATF5 expression was not consistently silenced epigenetically, therefore indicating that ATF5 is likely not a putative tumor suppressor in RCC.

## Hepatocellular carcinoma

Contrary to the oncogenic role of ATF5 in glioblastoma and other previously discussed malignancies, ATF5 appears to elicit a tumor suppressive role in hepatocellular carcinomas (HCCs). It has been reported that expression levels of ATF5 were significantly reduced in HCC relative to paired nonneoplastic hepatic tissue [51, 73]. ATF5 expression in HCC was also significantly associated with intrahepatic metastasis and liver cirrhosis, although there are mixed results pertaining to liver cirrhosis. Gho et al. showed that upon ectopic expression of ATF5, various HCC cell lines exemplified hindered growth up to 50% compared to controls with apoptosis ruled out as a confounding factor [51]. This effect on growth inhibition was reportedly due to cell cycle arrest at the G2-M phase of the cell cycle. Analysis of downstream targets of ATF5 via differential gene expression analysis revealed the helix-loop-helix protein ID1 as a target of ATF5 and mobility shift assays showed ATF5 binds to a cyclic AMP response element (CRE) within the promoter of ID1. Consistent with the repressive role of ATF5 at CREs, ATF5 expression was shown to inversely correlate with ID1 expression. Investigation into the mechanism of ATF5 downregulation in HCC indicated that DNA mutations, promoter methylation, histone modifications, and gene copy loss are all responsible for loss of ATF5 expression. Liu et al. reported that NPM1, a nucleolar chaperone, destabilizes ATF5 and that ectopic NPM1 expression abrogates ATF5-mediated effects on downstream targets in Hep3B cells [74]. In addition, the G2-M arrest and reduction in cell viability attributed to ectopic ATF5 expression was abrogated by co-expression with NPM1 in Hep3B cells. Wu et al. analyzed the prognostic significance of ATF5 expression in HCC, and found that high ATF5 expression was associated with recurrence-free survival in HCC hepatectomy patients but not overall survival [73]. Lastly, Gao et al. analyzed HCC samples paired to nonneoplastic hepatic tissue for differentially expressed- and methylated genes [75]. It was found that ATF5 promoter methylation was substantially increased in HCC and that ATF5 knockdown enhanced growth of various HCC cell lines, further supporting the tumor suppressive role of ATF5 in HCC.

Converse to the reported tumor suppressor role of ATF5 in HCC, Xu et al. report that ATF5 expression is regulated by miR-148a via decreased activation of the AKT/FOXO4/ATF5 pathway in HepG2 cells [76]. In addition, it was shown that ATF5 knockdown resulted in reduced mTOR protein levels. These findings suggest that although ATF5 inhibits the growth of HCC, it may still have the potential to possess an oncogenic role based on increased stimulation of AKT/mTOR pathways.

## Interference with ATF5 activity as a therapeutic strategy

As mentioned in the previous section on the role of ATF5 in cancer development, ATF5 possesses numerous properties that make it an attractive target for cancer treatment. The role of ATF5 in the transcriptional activation of proteins such as mTOR, BCL2, and MCL1 shows the importance of ATF5 in modulating activity of pathways critical to the development of cancer. Though this is true, ATF5 is a transcription factor and therefore is unlikely to be targetable via conventional small molecule targeting approaches. Initial use of a dominant-negative was developed as an experimental tool to modulate ATF5 activity in differentiation studies with neural progenitors [15]. The structure of this dominant-negative, termed Azip, is similar to that of WT ATF5 except the N-terminal portion is removed. This N-terminal portion includes the DNA-binding domain and acidic activation domain, thus eliminating the ability of this peptide to bind to DNA and participate in activation of transcription. This resulted in a dominant-negative protein that, in theory, binds to ATF5 and ATF5 binding partners to hinder their activity, but cannot partake in direct DNA binding. In addition, a region was added to the N-terminal portion of the protein called the enhanced leucine zipper which is composed of an acidic α-helix with leucines at every seventh residue. This enhanced leucine zipper has the potential to form salt bridges with the basic DNA binding domain of WT ATF5 or binding partners, thereby facilitating the intermolecular interactions between dnATF5 and WT ATF5. The C-terminal extended valine zipper region of ATF5 was also removed in order to reduce peptide aggregation [77].

**Figure 4:**
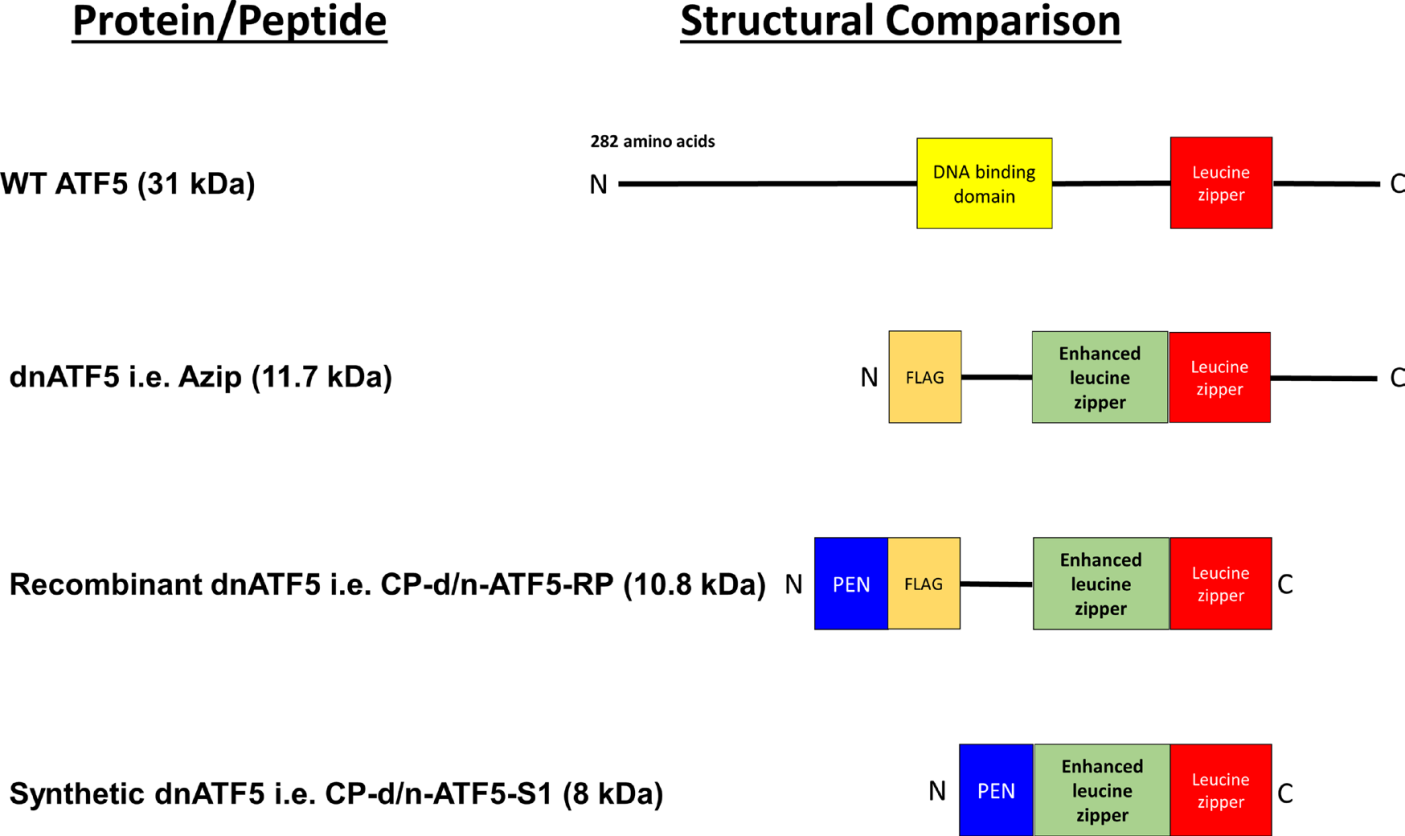
Comparison of WT ATF5 with dnATF5 Variants WT ATF5 contains a basic DNA binding domain adjacent to the leucine zipper domain. The first generation of dnATF5, i.e. Azip, was about a third of the molecular weight of WT ATF5, and had the N-terminal portion and the DNA binding domain removed, accompanied by an acidic enhanced leucine zipper and an N-terminal FLAG tag. This further led to the development of recombinant ATF5 which had the C-terminal portion of Azip removed and included the addition of an N-terminal penetratin motif. The synthetic variant of this cell-penetrant dnATF5 protein was further modified by removal of the FLAG tag and a segment of residual vector-derived amino acids. Image was generated using PowerPoint 2013 [80].

Further work led to the development of an Azip protein fused to a cell-penetrant protein, specifically a penetratin motif, thus allowing passage through the blood-brain barrier and extracellular entry into tumors upon systemic injection [55, 56, 78]. Two variants of this penetratin-fused dominant-negative were developed: a recombinant variant with a FLAG tag termed CP-d/n-ATF5-RP, and a synthetic variant termed CP-d/n-ATF5-S1. A structural comparison of WT ATF5, Azip, CP-d/n-ATF5-RP, and CP-d/n-ATF5-S1 can be seen in Figure 4.

## Future directions

The literature discussed in this review provides a foundation for the functional role of ATF5 in the context of cellular differentiation, stress, and cancer. Further studies would be useful in building upon this foundation. For example, utilizing conditional knockout models would be paramount to concluding the tissue-specific effects on differentiation mediated by ATF5. In addition, determining whether the functions of ATF5 in mitochondrial stress and the UPR^mt^ are relevant to cancer development would be critical toward elucidation of ATF5 function in cancer.

Developments in ATF5-targeted biologic therapies have resulted in the opening of an interesting new field of study. Here, small (< 10 kDa) dnATF5 peptides are injected systemically to elicit interference with ATF5 activity. Further advancements with this technology could include modifying the structure of the dominant-negative protein, such as altering the molecular weight or paying special attention to peptide sequences susceptible to proteolytic cleavage, to enhance the anticancer properties of this biologic. Altering the molecular weight of the peptide may alter its ability to cross cell membranes, therefore altering its distribution and clearance, whereas reduced proteolytic cleavage may result in decreased systemic elimination. In addition, studies should be performed to validate that dnATF5 is indeed targeting ATF5 and that the responses seen are not from an ATF5-independent action. The success of systemically deliverable dnATF5 peptides suggests this paradigm could be used in the development of therapeutic peptides targeting a wide variety of bZip transcription factors, such as AP-1. Further, a recent study by Huang et al. describes a novel approach to targeting ATF5 where apolipoprotein E3 high density lipoprotein is loaded with siRNA and calcium phosphate, and delivered to glioblastoma xenografts *in vivo* to inhibit tumor growth [79]. This also suggests gene therapy routes may be attractive delivery methods for ATF5-targeted therapies.

Previous studies show that ATF5 regulates neural stem cell differentiation and also cell fate in response to stress in adult neurons. Therefore, additional toxicity studies should be conducted to establish how dnATF5 administration may alter neural differentiation and survival of adult neurons.

## CONCLUSIONS

Dysregulation of ATF5 expression is commonly observed in a variety of different cancer types such as those of the brain, lung, and pancreas. Recent research has started to reveal the role of ATF5 in modulating various oncogenic characteristics such as evasion of apoptosis, tissue invasion, and deregulation of cellular bioenergetics. The critical role for ATF5 in the development of various cancer types has led to preclinical evaluation of several methods to target ATF5 and has yielded promising results. Future preclinical and clinical research will help reveal whether ATF5 is indeed a viable target for clinical cancer therapy.

## References

[1] Baxevanis AD, Vinson CR (1993). Interactions of coiled coils in transcription factors: where is the specificity?. Curr Opin Genet Dev.

[2] Ellenberger TE, Brandl CJ, Struhl K, Harrison SC (1992). The GCN4 basic region leucine zipper binds DNA as a dimer of uninterrupted α Helices: Crystal structure of the protein-DNA complex. Cell.

[3] Vinson C, Myakishev M, Acharya A, Mir AA, Moll JR, Bonovich M (2002). Classification of human B-ZIP proteins based on dimerization properties. Mol Cell Biol.

[4] Newman JR, Keating AE (2003). Comprehensive identification of human bZIP interactions with coiled-coil arrays. Science.

[5] Vattem KM, Wek RC (2004). Reinitiation involving upstream ORFs regulates ATF4 mRNA translation in mammalian cells. Proc Natl Acad Sci USA.

[6] Zhou D, Palam LR, Jiang L, Narasimhan J, Staschke KA, Wek RC (2008). Phosphorylation of eIF2 Directs ATF5 Translational Control in Response to Diverse Stress Conditions. J Biol Chem.

[7] Quirós PM, Prado MA, Zamboni N, D’Amico D, Williams RW, Finley D, Gygi SP, Auwerx J (2017). Multi-omics analysis identifies ATF4 as a key regulator of the mitochondrial stress response in mammals. J Cell Biol.

[8] Galehdar Z, Swan P, Fuerth B, Callaghan SM, Park DS, Cregan SP (2010). Neuronal apoptosis induced by endoplasmic reticulum stress is regulated by ATF4–CHOP-mediated induction of the Bcl-2 homology 3-only member PUMA. J Neurosci.

[9] Fels DR, Koumenis C (2006). The PERK/eIF2α/ATF4 module of the UPR in hypoxia resistance and tumor growth. Cancer Biol Ther.

[10] Watatani Y, Ichikawa K, Nakanishi N, Fujimoto M, Takeda H, Kimura N, Hirose H, Takahashi S, Takahashi Y (2008). Stress-induced translation of ATF5 mRNA is regulated by the 5′-untranslated region. J Biol Chem.

[11] Hatano M, Umemura M, Kimura N, Yamazaki T, Takeda H, Nakano H, Takahashi S, Takahashi Y (2013). The 5′-untranslated region regulates ATF5 mRNA stability via nonsense-mediated mRNA decay in response to environmental stress. FEBS J.

[12] Hansen MB, Mitchelmore C, Kjaerulf KM, Rasmussen TE, Pedersen KM, Jensen NA (2002). Mouse Atf5: molecular cloning of two novel mRNAs, genomic organization, and odorant sensory neuron localization. Genomics.

[13] Pati D, Meistrich ML, Plon SE (1999). Human Cdc34 and Rad6B ubiquitin-conjugating enzymes target repressors of cyclic AMP-induced transcription for proteolysis. Mol Cell Biol.

[14] Wei Y, Jiang J, Liu D, Zhou J, Chen X, Zhang S, Zong H, Yun X, Gu J (2008). Cdc34-mediated degradation of ATF5 is blocked by cisplatin. J Biol Chem.

[15] Angelastro JM, Ignatova TN, Kukekov VG, Steindler DA, Stengren GB, Mendelsohn C, Greene LA (2003). Regulated expression of ATF5 is required for the progression of neural progenitor cells to neurons. J Neurosci.

[16] Leong DT, Abraham MC, Gupta A, Lim TC, Chew FT, Hutmacher DW (2012). ATF5, a possible regulator of osteogenic differentiation in human adipose-derived stem cells. J Cell Biochem.

[17] Nakamori D, Takayama K, Nagamoto Y, Mitani S, Sakurai F, Tachibana M, Mizuguchi H (2016). Hepatic maturation of human iPS cell-derived hepatocyte-like cells by ATF5, c/EBPα, and PROX1 transduction. Biochem Biophys Res Commun.

[18] Zhao Y, Zhang YD, Zhang YY, Qian SW, Zhang ZC, Li SF, Guo L, Liu Y, Wen B, Lei QY, Tang QQ, Li X (2014). p300-dependent acetylation of activating transcription factor 5 enhances C/EBPβ transactivation of C/EBPα during 3T3-L1 differentiation. Mol Cell Biol.

[19] Nakano H, Iida Y, Suzuki M, Aoki M, Umemura M, Takahashi S, Takahashi Y (2016). Activating transcription factor 5 (ATF5) is essential for the maturation and survival of mouse basal vomeronasal sensory neurons. Cell Tissue Res.

[20] Wang SZ, Ou J, Zhu LJ, Green MR (2012). Transcription factor ATF5 is required for terminal differentiation and survival of olfactory sensory neurons. Proc Natl Acad Sci USA.

[21] Angelastro JM, Klimaschewski L, Tang S, Vitolo OV, Weissman TA, Donlin LT, Shelanski ML, Greene LA (2000). Identification of diverse nerve growth factor-regulated genes by serial analysis of gene expression (SAGE) profiling. Proc Natl Acad Sci USA.

[22] Angelastro JM, Mason JL, Ignatova TN, Kukekov VG, Stengren GB, Goldman JE, Greene LA (2005). Downregulation of activating transcription factor 5 is required for differentiation of neural progenitor cells into astrocytes. J Neurosci.

[23] Mason JL, Angelastro JM, Ignatova TN, Kukekov VG, Lin G, Greene LA, Goldman JE (2005). ATF5 regulates the proliferation and differentiation of oligodendrocytes. Mol Cell Neurosci.

[24] Lee HY, Angelastro JM, Kenney AM, Mason CA, Greene LA (2012). Reciprocal actions of ATF5 and Shh in proliferation of cerebellar granule neuron progenitor cells. Dev Neurobiol.

[25] Torres-Peraza JF, Engel T, Martin-Ibanez R, Sanz-Rodriguez A, Fernandez-Fernandez MR, Esgleas M, Canals JM, Henshall DC, Lucas JJ (2013). Protective neuronal induction of ATF5 in endoplasmic reticulum stress induced by status epilepticus. Brain.

[26] Karpel-Massler G, Horst BA, Shu C, Chau L, Tsujiuchi T, Bruce JN, Canoll P, Greene LA, Angelastro JM, Siegelin MD (2016). A Synthetic Cell-Penetrating Dominant-Negative ATF5 Peptide Exerts Anticancer Activity against a Broad Spectrum of Treatment-Resistant Cancers. Clin Cancer Res.

[27] Arias A, Lamé MW, Santarelli L, Hen R, Greene LA, Angelastro JM (2012). Regulated ATF5 loss-of-function in adult mice blocks formation and causes regression/eradication of gliomas. Oncogene.

[28] Du Y, Wang J, Jia J, Song N, Xiang C, Xu J, Hou Z, Su X, Liu B, Jiang T, Zhao D, Sun Y, Shu J (2014). Human Hepatocytes with Drug Metabolic Function Induced from Fibroblasts by Lineage Reprogramming. Cell Stem Cell.

[29] Pascual M, Gomez-Lechon MJ, Castell JV, Jover R (2008). ATF5 is a highly abundant liver-enriched transcription factor that cooperates with constitutive androstane receptor in the transactivation of CYP2B6: implications in hepatic stress responses. Drug Metab Dispos.

[30] Fiorese CJ, Schulz AM, Lin YF, Rosin N, Pellegrino MW, Haynes CM (2016). The transcription factor ATF5 mediates a mammalian mitochondrial UPR. Curr Biol.

[31] Wang H, Lin G, Zhang Z (2007). ATF5 promotes cell survival through transcriptional activation of Hsp27 in H9c2 cells. Cell Biol Int.

[32] Juliana CA, Yang J, Rozo AV, Good A, Groff DN, Wang SZ, Green MR, Stoffers DA (2017). ATF5 regulates beta-cell survival during stress. Proc Natl Acad Sci USA.

[33] Dluzen D, Li G, Tacelosky D, Moreau M, Liu DX (2011). BCL-2 Is a Downstream Target of ATF5 That Mediates the Prosurvival Function of ATF5 in a Cell Type-dependent Manner. J Biol Chem.

[34] Sheng Z, Li L, Zhu LJ, Smith TW, Demers A, Ross AH, Moser RP, Green MR (2010). A genome-wide RNA interference screen reveals an essential CREB3L2-ATF5-MCL1 survival pathway in malignant glioma with therapeutic implications. Nat Med.

[35] Ito H, Iwamoto I, Inaguma Y, Takizawa T, Nagata K, Asano T, Kato K (2005). Endoplasmic reticulum stress induces the phosphorylation of small heat shock protein, Hsp27. J Cell Biochem.

[36] Watterson TL, Hamilton B, Martin R, Coulombe RA (2009). Urban Particulate Matter Causes ER Stress and the Unfolded Protein Response in Human Lung Cells. Toxicol Sci.

[37] Li G, Xu Y, Guan D, Liu Z, Liu DX (2011). HSP70 protein promotes survival of C6 and U87 glioma cells by inhibition of ATF5 degradation. J Biol Chem.

[38] Izumi S, Saito A, Kanemoto S, Kawasaki N, Asada R, Iwamoto H, Oki M, Miyagi H, Ochi M, Imaizumi K (2012). The endoplasmic reticulum stress transducer BBF2H7 suppresses apoptosis by activating the ATF5-MCL1 pathway in growth plate cartilage. J Biol Chem.

[39] Teske BF, Fusakio ME, Zhou D, Shan J, McClintick JN, Kilberg MS, Wek RC (2013). CHOP induces activating transcription factor 5 (ATF5) to trigger apoptosis in response to perturbations in protein homeostasis. Mol Biol Cell.

[40] Yamazaki T, Ohmi A, Kurumaya H, Kato K, Abe T, Yamamoto H, Nakanishi N, Okuyama R, Umemura M, Kaise T, Watanabe R, Okawa Y, Takahashi S, Takahashi Y (2010). Regulation of the human CHOP gene promoter by the stress response transcription factor ATF5 via the AARE1 site in human hepatoma HepG2 cells. Life Sci.

[41] Jonas JC, Bensellam M, Duprez J, Elouil H, Guiot Y, Pascal SM (2009). Glucose regulation of islet stress responses and beta-cell failure in type 2 diabetes. Diabetes Obes Metab.

[42] Sachdeva MM, Claiborn KC, Khoo C, Yang J, Groff DN, Mirmira RG, Stoffers DA (2009). Pdx1 (MODY4) regulates pancreatic beta cell susceptibility to ER stress. Proc Natl Acad Sci USA.

[43] Oslowski CM, Hara T, O’Sullivan-Murphy B, Kanekura K, Lu S, Hara M, Ishigaki S, Zhu LJ, Hayashi E, Hui ST, Greiner D, Kaufman RJ, Bortell R, Urano F (2012). Thioredoxin-Interacting Protein Mediates ER Stress-Induced β Cell Death through Initiation of the Inflammasome. Cell Metab.

[44] Zhao Q, Wang J, Levichkin IV, Stasinopoulos S, Ryan MT, Hoogenraad NJ (2002). A mitochondrial specific stress response in mammalian cells. EMBO J.

[45] Mancini C, Roncaglia P, Brussino A, Stevanin G, Lo Buono N, Krmac H, Maltecca F, Gazzano E, Bartoletti Stella A, Calvaruso MA, Iommarini L, Cagnoli C, Forlani S (2013). Genome-wide expression profiling and functional characterization of SCA28 lymphoblastoid cell lines reveal impairment in cell growth and activation of apoptotic pathways. BMC Med Genomics.

[46] Deng P, Haynes CM (2017). Mitochondrial dysfunction in cancer: Potential roles of ATF5 and the mitochondrial UPR. Semin Cancer Biol.

[47] Reinke AW, Baek J, Ashenberg O, Keating AE (2013). Networks of bZIP Protein-Protein Interactions Diversified Over a Billion Years of Evolution. Science.

[48] Kong X, Meng W, Zhou Z, Li Y, Zhou B, Wang R, Zhan L (2011). Overexpression of activating transcription factor 5 in human rectal cancer. Exp Ther Med.

[49] Hua XM, Wang J, Qian DM, Song JY, Chen H, Zhu XL, Zhou R, Zhao YD, Zhou XZ, Li L, Zhang L, Song XX, Wang B (2015). DNA methylation level of promoter region of activating transcription factor 5 in glioma. J Zhejiang Univ Sci B.

[50] Madarampalli B, Yuan Y, Liu D, Lengel K, Xu Y, Li G, Yang J, Liu X, Lu Z, Liu DX (2015). ATF5 Connects the Pericentriolar Materials to the Proximal End of the Mother Centriole. Cell.

[51] Gho JW, Ip WK, Chan KY, Law PT, Lai PB, Wong N (2008). Re-expression of transcription factor ATF5 in hepatocellular carcinoma induces G2-M arrest. Cancer Res.

[52] Dong S, Nutt CL, Betensky RA, Stemmer-Rachamimov AO, Denko NC, Ligon KL, Rowitch DH, Louis DN (2005). Histology-Based Expression Profiling Yields Novel Prognostic Markers in Human Glioblastoma. J Neuropathol Exp Neurol.

[53] Huang R, Qian D, Hu M, Zhang X, Song J, Li L, Chen H, Wang B (2015). Association between human cytomegalovirus infection and histone acetylation level in various histological types of glioma. Oncol Lett.

[54] Angelastro JM, Canoll PD, Kuo J, Weicker M, Costa A, Bruce JN, Greene LA (2006). Selective destruction of glioblastoma cells by interference with the activity or expression of ATF5. Oncogene.

[55] Cates CC, Arias AD, Nakayama Wong LS, Lamé MW, Sidorov M, Cayanan G, Rowland DJ, Fung J, Karpel-Massler G, Siegelin MD, Greene LA, Angelastro JM (2016). Regression/eradication of gliomas in mice by a systemically-deliverable ATF5 dominant-negative peptide. Oncotarget.

[56] Karpel-Massler G, Horst BA, Shu C, Chau L, Tsujiuchi T, Bruce JN, Canoll P, Greene LA, Angelastro JM, Siegelin MD (2016). A synthetic cell-penetrating dominant-negative ATF5 peptide exerts anticancer activity against a broad spectrum of treatment-resistant cancers. Clin Cancer Res.

[57] Delgado-Lopez PD, Corrales-Garcia EM (2016). Survival in glioblastoma: a review on the impact of treatment modalities. Clin Transl Oncol.

[58] Monaco SE, Angelastro JM, Szabolcs M, Greene LA (2007). The transcription factor ATF5 is widely expressed in carcinomas, and interference with its function selectively kills neoplastic, but not nontransformed, breast cell lines. Int J Cancer.

[59] Li G, Li W, Angelastro JM, Greene LA, Liu DX (2009). Identification of a novel DNA binding site and a transcription target for ATF5 in C6 glioma and MCF-7 breast cancer cells. Mol Cancer Res.

[60] Nukuda A, Endoh H, Yasuda M, Mizutani T, Kawabata K, Haga H (2016). Role of ATF5 in the invasive potential of diverse human cancer cell lines. Biochem Biophys Res Commun.

[61] Devireddy LR, Teodoro JG, Richard FA, Green MR (2001). Induction of apoptosis by a secreted lipocalin that is transcriptionally regulated by IL-3 deprivation. Science.

[62] Persengiev SP, Devireddy LR, Green MR (2002). Inhibition of apoptosis by ATFx: a novel role for a member of the ATF/CREB family of mammalian bZIP transcription factors. Genes Dev.

[63] Sheng Z, Ma L, Sun JE, Zhu LJ, Green MR (2011). BCR-ABL suppresses autophagy through ATF5-mediated regulation of mTOR transcription. Blood.

[64] Rousseau J, Gagne V, Labuda M, Beaubois C, Sinnett D, Laverdiere C, Moghrabi A, Sallan SE, Silverman LB, Neuberg D, Kutok JL, Krajinovic M (2011). ATF5 polymorphisms influence ATF function and response to treatment in children with childhood acute lymphoblastic leukemia. Blood.

[65] Mittal AK, Hegde GV, Aoun P, Bociek RG, Dave BJ, Joshi AD, Sanger WG, Weisenburger DD, Joshi SS (2007). Molecular basis of aggressive disease in chronic lymphocytic leukemia patients with 11q deletion and trisomy 12 chromosomal abnormalities. Int J Mol Med.

[66] Montoto S, Davies AJ, Matthews J, Calaminici M, Norton AJ, Amess J, Vinnicombe S, Waters R, Rohatiner AZ, Lister TA (2007). Risk and clinical implications of transformation of follicular lymphoma to diffuse large B-cell lymphoma. J Clin Oncol.

[67] Ishihara S, Yasuda M, Ishizu A, Ishikawa M, Shirato H, Haga H (2015). Activating transcription factor 5 enhances radioresistance and malignancy in cancer cells. Oncotarget.

[68] Fernandez P, Carretero J, Medina PP, Jimenez AI, Rodriguez-Perales S, Paz MF, Cigudosa JC, Esteller M, Lombardia L, Morente M, Sanchez-Verde L, Sotelo T, Sanchez-Cespedes M (2004). Distinctive gene expression of human lung adenocarcinomas carrying LKB1 mutations. Oncogene.

[69] Chen A, Qian D, Wang B, Hu M, Lu J, Qi Y, Liu DX (2012). ATF5 is overexpressed in epithelial ovarian carcinomas and interference with its function increases apoptosis through the downregulation of Bcl-2 in SKOV-3 cells. Int J Gynecol Pathol.

[70] Hu M, Wang B, Qian D, Li L, Zhang L, Song X, Liu DX (2012). Interference with ATF5 function enhances the sensitivity of human pancreatic cancer cells to paclitaxel-induced apoptosis. Anticancer Res.

[71] Ye XJ, Zhang ZW, Lin GT (2003). Expression of activating transcription factor 5 (ATF5) in renal cell carcinoma. Chinese J Urology.

[72] Morris MR, Gentle D, Abdulrahman M, Clarke N, Brown M, Kishida T, Yao M, Teh BT, Latif F, Maher ER (2008). Functional epigenomics approach to identify methylated candidate tumour suppressor genes in renal cell carcinoma. Br J Cancer.

[73] Wu Y, Wu B, Chen R, Zheng Y, Huang Z (2014). High ATF5 expression is a favorable prognostic indicator in patients with hepatocellular carcinoma after hepatectomy. Med Oncol.

[74] Liu X, Liu D, Qian D, Dai J, An Y, Jiang S, Stanley B, Yang J, Wang B, Liu X (2012). Nucleophosmin (NPM1/B23) interacts with activating transcription factor 5 (ATF5) protein and promotes proteasome-and caspase-dependent ATF5 degradation in hepatocellular carcinoma cells. J Biol Chem.

[75] Gao F, Xia Y, Wang J, Lin Z, Ou Y, Liu X, Liu W, Zhou B, Luo H, Zhou B, Wen B, Zhang X, Huang J (2014). Integrated analyses of DNA methylation and hydroxymethylation reveal tumor suppressive roles of ECM1, ATF5, and EOMES in human hepatocellular carcinoma. Genome Biol.

[76] Xu X, Fan Z, Kang L, Han J, Jiang C, Zheng X, Zhu Z, Jiao H, Lin J, Jiang K, Ding L, Zhang H, Cheng L (2013). Hepatitis B virus X protein represses miRNA-148a to enhance tumorigenesis. J Clin Invest.

[77] Ciaccio NA, Reynolds TS, Middaugh CR, Laurence JS (2012). Influence of the Valine Zipper Region on the Structure and Aggregation of the Basic Leucine Zipper (bZIP) Domain of Activating Transcription Factor 5 (ATF5). Mol Pharm.

[78] Copolovici DM, Langel K, Eriste E, Langel Ü (2014). Cell-Penetrating Peptides: Design, Synthesis, and Applications. ACS Nano.

[79] Huang JL, Jiang G, Song QX, Gu X, Hu M, Wang XL, Song HH, Chen LP, Lin YY, Jiang D, Chen J, Feng JF, Qiu YM (2017). Lipoprotein-biomimetic nanostructure enables efficient targeting delivery of siRNA to Ras-activated glioblastoma cells via macropinocytosis. Nat Commun.

[80] Angelastro JM (2017). Targeting ATF5 in Cancer. Trends Cancer.

